# Host cell depletion of tryptophan by IFNγ-induced Indoleamine 2,3-dioxygenase 1 (IDO1) inhibits lysosomal replication of *Coxiella burnetii*

**DOI:** 10.1371/journal.ppat.1007955

**Published:** 2019-08-28

**Authors:** Sandhya Ganesan, Craig R. Roy

**Affiliations:** Department of Microbial Pathogenesis, Boyer Center for Molecular Medicine, Yale University School of Medicine, New Haven, Connecticut, United States of America; Purdue University, UNITED STATES

## Abstract

Most intracellular pathogens that reside in a vacuole prevent transit of their compartment to lysosomal organelles. Effector mechanisms induced by the pro-inflammatory cytokine Interferon-gamma (IFNγ) can promote the delivery of pathogen-occupied vacuoles to lysosomes for proteolytic degradation and are therefore important for host defense against intracellular pathogens. The bacterial pathogen *Coxiella burnetii* is unique in that, transport to the lysosome is essential for replication. The bacterium modulates membrane traffic to create a specialized autophagolysosomal compartment called the *Coxiella*-containing vacuole (CCV). Importantly, IFNγ signaling inhibits intracellular replication of *C*. *burnetii*, raising the question of which IFNγ-activated mechanisms restrict replication of a lysosome-adapted pathogen. To address this question, siRNA was used to silence a panel of IFNγ-induced genes in HeLa cells to identify genes required for restriction of *C*. *burnetii* intracellular replication. This screen demonstrated that Indoleamine 2,3-dioxygenase 1 (IDO1) contributes to IFNγ-mediated restriction of *C*. *burnetii*. IDO1 is an enzyme that catabolizes cellular tryptophan to kynurenine metabolites thereby reducing tryptophan availability in cells. Cells deficient in IDO1 function were more permissive for *C*. *burnetii* replication when treated with IFNγ, and supplementing IFNγ-treated cells with tryptophan enhanced intracellular replication. Additionally, ectopic expression of IDO1 in host cells was sufficient to restrict replication of *C*. *burnetii* in the absence of IFNγ signaling. Using differentiated THP1 macrophage-like cells it was determined that IFNγ-activation resulted in IDO1 production, and that supplementation of IFNγ-activated THP1 cells with tryptophan enhanced *C*. *burnetii* replication. Thus, this study identifies IDO1 production as a key cell-autonomous defense mechanism that limits infection by *C*. *burnetii*, which suggests that peptides derived from hydrolysis of proteins in the CCV do not provide an adequate supply of tryptophan for bacterial replication.

## Author summary

*Coxiella burnetii* is a mammalian pathogen that can cause a predominantly zoonotic disease called Q-fever. In humans, Q-fever manifests as an acute or chronic illness especially in immunocompromised individuals. *C*. *burnetii* is uniquely adapted to live in a lysosome-derived vacuole that degrades proteins and provides nutrients that support intracellular replication. From a cell biological perspective, *C*. *burnetii* represents an excellent model to study pathogens that survive in harsh cellular environments. The strategies by which infected cells intrinsically combat *C*. *burnetii* are not well-established. In this study, we investigate the underlying mechanism by which IFNγ activates cells and prevents *C*. *burnetii* from replicating inside cells. The data presented here demonstrate that IFNγ induces the expression of the enzyme Indoleamine 2,3-dioxygenase 1 (IDO1), which degrades the amino acid tryptophan and restricts the intracellular replication of *C*. *burnetii*. The production of IDO1 is sufficient to inhibit replication of *C*. *burnetii*, indicating that tryptophan depletion is an effective cell-autonomous defense mechanism against this lysosome-adapted pathogen. In addition, these data imply that the degradative vacuole in which this pathogen resides does not generate a supply of tryptophan sufficient to support intracellular replication.

## Introduction

*Coxiella burnetii* is a gram-negative, obligate intracellular pathogen that causes an infectious disease called Q-fever. Humans are occasionally infected through inhalation of aerosols or through close contact with infected livestock, and the symptoms range from mild flu-like illness to vascular complications and fatal endocarditis (reviewed in [1]). Infection of human cells begins with the phagocytosis of *C*. *burnetii*. Phagosomes containing *C*. *burnetii* undergo endocytic maturation and fuse with lysosomes, which results in the formation of the *Coxiella-*containing vacuole (CCV). Acidification of the CCV activates the *C*. *burnetii* Type IVB secretion system (T4SS) called Dot/Icm, which promotes the translocation of roughly 100 different bacterial effector proteins into the host cell cytosol [2,3]. Type IV secretion is essential for intracellular replication of *C*. *burnetii* and the generation of a spacious CCV that has autophagolysosomal characteristics [3–5]. Individual Type IV effector proteins (T4E) facilitate evasion of innate immune surveillance and acquisition of nutrients and membrane for the CCV (reviewed in [6]). The development of an axenic culture medium and genetic manipulation techniques have made *C*. *burnetii* an excellent system to study how pathogens adapt to survive and replicate in a lysosome-derived organelle as well as the cell-autonomous immune strategies in place to control their intracellular replication [7,8].

Adaptive immune responses lead to the production of IFNγ, which is a critical determinant of host protection against *C*. *burnetii* in immunocompetent animals [9,10]. IFNγ is a potent pro-inflammatory cytokine secreted by activated lymphocytes during infection. Circulating IFNγ has been reported to be a sensitive and diagnostic biomarker in Q fever patients, which shows that an adaptive cell-mediated immune response has been generated [11,12]. IFNγ receptors, ubiquitously expressed on various cell types, bind to IFNγ and stimulate the Janus kinase-Signal transducer and activator of transcription (JAK-STAT) signaling cascade that activates expression of hundreds of antimicrobial genes that provide cell-autonomous defense against intracellular pathogens. The functions of IFNγ-induced genes include, but are not restricted to, generation of reactive oxygen and nitrogen radicals, antimicrobial peptides, toxic metabolites, activation of immune signaling, immunoproteasome, antigen presentation, vesicle traffic, autophagy, immune GTPases, small molecule transporters and production of soluble messengers such as cytokines and chemokines (reviewed in [13]). IFNγ-mediated elimination of intravacuolar pathogens (e.g. *Salmonella*, *Mycobacteria*) involves immune GTPase and autophagic-recognition of the pathogen-containing compartment (PCV) and labeling it for lysosomal fusion and degradation [14–16]. In the case of pathogens which rupture their phagosomal vacuole and escape to the cytosol (e.g. *Listeria*), autophagic response triggers the delivery of the bacteria to the lysosome [17,18]. IFNγ-mediated restriction of *C*. *burnetii* replication in professional phagocytic cells has been attributed to phenotypes that include CCV alkalinization, TNF-mediated apoptosis, and generation of reactive nitrogen and oxygen species [19–23]. However, restriction mechanisms against pathogens that have evolved to survive and replicate in hostile lysosomal compartments have not been extensively characterized.

In an effort to identify and characterize specific host proteins that are induced in IFNγ-activated cells and participate in the restriction of *C*. *burnetii* intracellular replication, an siRNA screen using a curated set of IFNγ-induced genes was conducted. Data from the screen shows that Indoleamine 2,3-dioxygenase 1 (IDO1) is an IFNγ-induced effector that contributes to the restriction of *C*. *burnetii* intracellular replication. IDO1 is an enzyme that catalyzes the conversion of the essential amino acid L-tryptophan to kynurenines, which are then used for the synthesis of the metabolite nicotinamide adenine dinucleotide (NAD+) (reviewed in [24]). Because *C*. *burnetii* is a tryptophan auxotroph [25], these data show that one mechanism by which IFNγ restricts the replication of this intracellular pathogen is through IDO1-mediated depletion of an essential nutrient.

## Results

### IFNγ restricts *C*. *burnetii* intracellular replication, CCV size, effector translocation and bacterial infectivity

**Fig 1:**
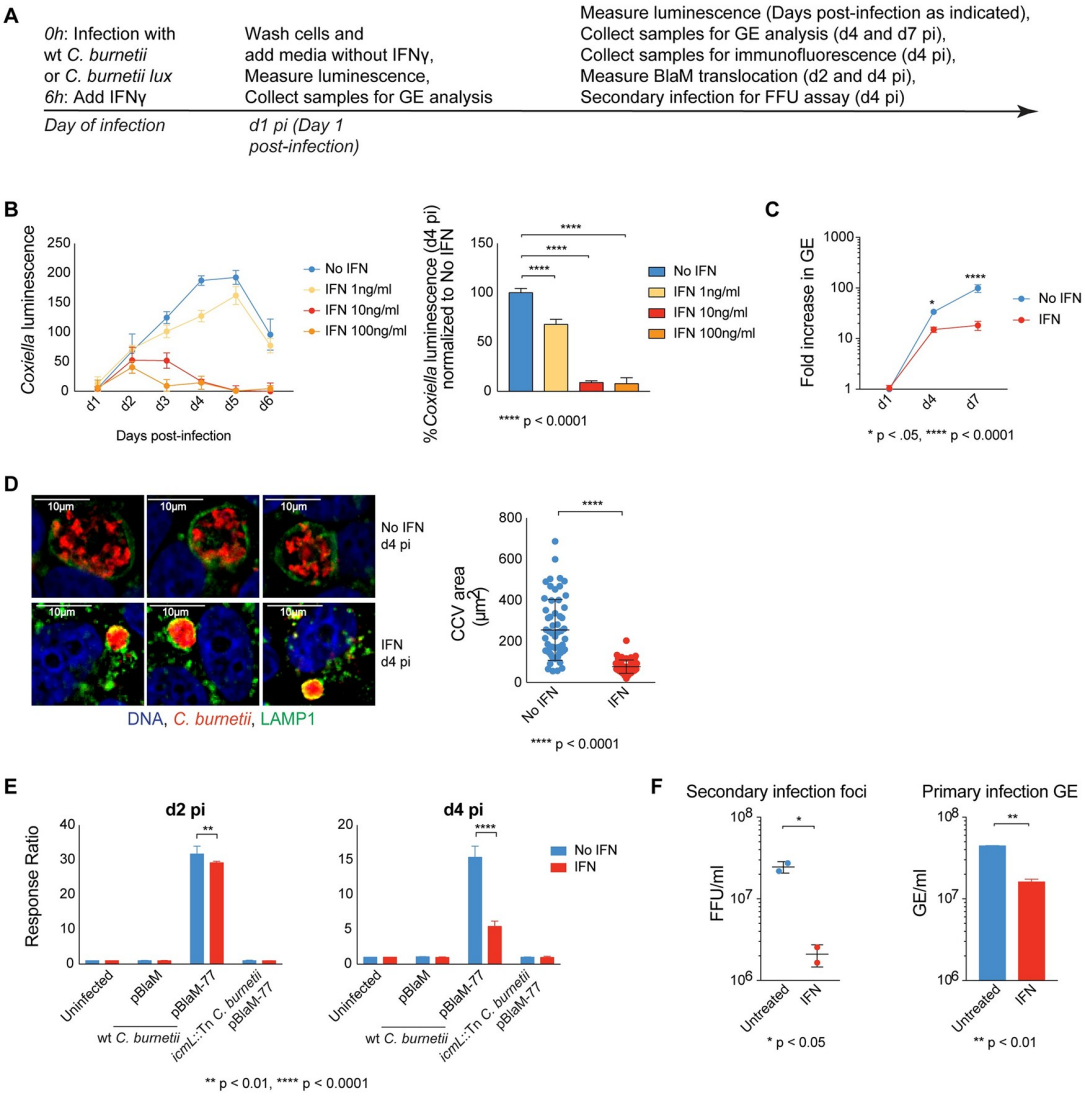
IFNγ restricts *C*. *burnetii* intracellular replication, CCV size, effector translocation and bacterial infectivity. Experimental setup is laid out in A. HeLa cells were infected with *C*. *burnetii lux* (B) or wt *C*. *burnetii* (C-F) and treated with recombinant IFNγ at 10ng/ml (C-F) or as indicated (B). *C*. *burnetii* replication was monitored by measuring luminescence values on indicated days post-infection (B) or by fold increase in genome equivalents (GE) on d4 and d7 pi compared to that of d1 pi (C). CCV and the intracellular bacteria were visualized by indirect immunofluorescence with anti-LAMP1 and anti-*C*. *burnetii* antibodies on d4 pi (D). Average CCV area for approximately 50 vacuoles from different fields were quantified per treatment and averaged (D). HeLa cells were infected with *C*. *burnetii* pBlaM or pBlaM-77 and left untreated or treated with IFNγ. On d2 and d4 pi, cells were loaded with the fluorescent substrate CCF4-AM and translocation of BlaM was assessed by the shift in the fluorescence emission from 535 to 460nm relative to that of uninfected (E). Focus forming units (FFU) of bacteria obtained from HeLa cells treated with or without IFNγ were determined by secondary infection of untreated HeLa cells and counting of *C*. *burnetii*-stained foci on d4 pi (F). Panel to the right shows GE obtained from primary infection (F).

Macrophages treated with IFNγ will restrict *C*. *burnetii* replication by a process that is mediated in part by production of inducible nitric oxide synthase and NADPH oxidase [21–23]. Data demonstrating that macrophages deficient in these enzymes still robustly restrict *C*. *burnetii* replication indicates that there must be multiple mechanisms by which mammalian cells restrict intracellular replication of *C*. *burnetii* upon stimulation by IFNγ [23]. To identify additional pathways by which IFNγ stimulation restricts intracellular replication of *C*. *burnetii*, we examined whether treatment of HeLa 229 cells would restrict intracellular replication of this pathogen. The rationale for using HeLa cells was that these cells have many evolutionarily conserved antimicrobial mechanisms that can restrict the replication of intracellular bacterial pathogens (reviewed in [26]), but HeLa cells may not have as many independent pathways to limit the intracellular replication of *C*. *burnetii* upon IFNγ treatment as macrophages given that these are human derived epithelial cells and not professional phagocytes. This would increase the likelihood of identifying host factors important for growth restriction by reducing the possibility of redundancy. In addition, HeLa cells are amenable to genetic manipulation. A layout of the experimental setup is presented in Fig 1A. Luminescence generated by a *C*. *burnetii* strain expressing the *luxCDABE* operon constitutively, served as an indicator of bacterial replication (Fig 1B). HeLa cells were infected with *C*. *burnetii* and treated with increasing concentrations of IFNγ at 6h post infection (pi). At concentrations as low as 10 ng/ml, there was a significant decrease in the luminescence values observed as early as day 3 (d3) pi. Based on these data, d4 pi was used as the standard time-point for subsequent experiments measuring bacterial luminescence (Fig 1B). In agreement with luminescence readouts, *C*. *burnetii* genome equivalents (GE) measured by quantitative PCR also showed a significant decline in bacterial numbers from IFNγ-treated cells (Fig 1C). Of note, GE and luminescence data are graphed on log and linear scales respectively, yet reveal the same trend. Reduced bacterial numbers in IFNγ-treated cells also correlated with smaller CCV sizes on d4 pi (Fig 1D). PFA-fixed cells stained for LAMP1 and *C*. *burnetii* were visualized by indirect immunofluorescence microscopy. The CCVs in IFNγ-treated cells were small and the bacteria were packed tightly, whereas, untreated cells had larger and more spacious CCVs that contained dispersed bacteria (Fig 1D). Quantification showed that the average CCV size in IFNγ-treated cells was reduced significantly compared to that of untreated cells (Fig 1D).

Effector proteins translocated by the Dot/Icm secretion system are required to subvert host vesicle traffic to promote the expansion of the CCV. The observation that the CCVs were smaller in IFNγ-treated cells raised the question of whether IFNγ signaling interferes with the ability of *C*. *burnetii* to translocate effector proteins. Examination of Dot/Icm-dependent translocation of the effector proteins was measured using the effector Cbu0077 fused to the translocation reporter BlaM encoding a β-lactamase enzyme that will cleave the fluorescent substrate loaded into host cells. These data demonstrated a significant reduction in BlaM-Cbu0077 translocation in IFNγ-treated cells on d2 pi and on d4 pi (Fig 1E). Because unified CCVs were observed in IFNγ-treated cells, which is a phenotype requiring the effector protein Cig2 [27], it is likely that *C*. *burnetii* are initially capable of translocating early effector proteins in cells that were treated with IFNγ 6h after infection. However, the decrease in translocation of BlaM-Cbu0077 observed at d2 and d4 pi indicates that IFNγ-treatment leads to inhibition of Dot/Icm function at these later times, which is consistent with the decrease observed in bacterial luminescence and replication (Fig 1E).

To determine if *C*. *burnetii* isolated from IFNγ-treated cells were viable and capable of initiating a secondary infection, a foci-forming unit (FFU) assay was performed. Lysates and supernatants of *C*. *burnetii*-infected cells that were either untreated or treated with IFNγ were collected on d4 pi. Dilutions of each sample were used to measure the number of bacteria by quantitative PCR (GE) and infect untreated HeLa cells. Infected cells were fixed and stained using an anti-*C*. *burnetii* antibody on d4 pi and immunofluorescence microscopy was used to determine the FFU value after counting the number of cells containing large CCVs (Fig 1F, left panel). *C*. *burnetii* collected from untreated cells (primary infection) gave rise to a FFU value that was almost a log higher than that isolated from IFNγ-treated cells infected in parallel. This difference was comparatively larger than that obtained using the GE assay (Fig 1F, right panel). This indicates that IFNγ treatment reduces the viability of *C*. *burnetii* that were still detectable using the GE assay. Together, these data demonstrate that IFNγ limits the expansion of the CCV, intracellular replication, metabolic activity and viability of *C*. *burnetii* by inducing cell-autonomous defense mechanisms. Thus, in addition to restricting *C*. *burnetii* in professional phagocytes [19,20,22,23], these results indicate that IFNγ induces antimicrobial activities that are capable of restricting *C*. *burnetii* replication in cells that are normally non-phagocytic, which suggests there could be a shared IFNγ-activated antimicrobial activity in these cells.

### Loss of function analysis of IFNγ-induced genes to identify host factors that mediate restriction of *C*. *burnetii* replication

**Fig 2:**
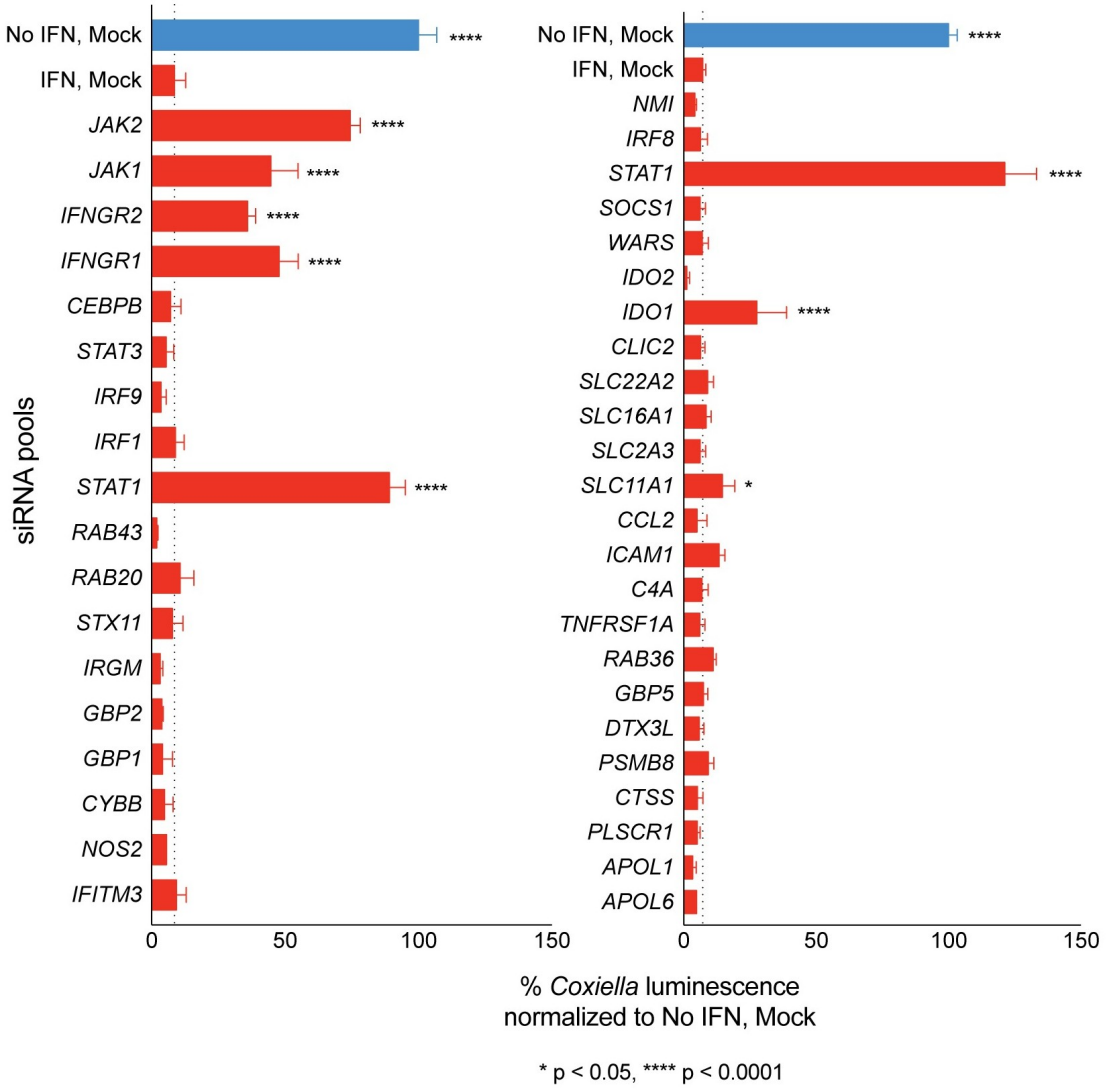
Loss of function analysis of IFNγ-induced genes to identify host factors that mediate restriction of *C*. *burnetii* replication. Two panels of selected IFNγ-induced genes were silenced by siRNA in HeLa cells. Cells were infected with *C*. *burnetii lux* and left untreated (blue bars) or treated with IFNγ (red bars) at 6h pi. Luminescence values measured on d4 pi were normalized to that of untreated, mock-transfected cells (blue bars). Genes that function in the IFNγ signaling pathway (IFNγ receptors, JAK kinases and the STAT1 transcription factor) were included as positive controls. Dotted lines indicate % luminescence values for mock-transfected, IFNγ-treated cells, which was used as the baseline to calculate statistical significance for each set.

Depending on the cell type, IFNγ signaling stimulates the expression of hundreds to thousands of genes. Published data sets profiling genes upregulated by IFNγ in HeLa cells and macrophages were used to curate genes that may be involved in restricting *C*. *burnetii* replication [28–31]. Genes encoding proteins that regulate transport and fusion of membranes were given high priority, as were regulators of nutrient transport and cell metabolism. To identify the specific host factors that mediate restriction, a subset of genes upregulated following IFNγ treatment was silenced individually using siRNA. Cell-surface receptors for the cytokine IFNγ (IFNγR1 and IFNγR2), which signal the kinases of the JAK-STAT signal transduction pathway (JAK1, JAK2) to activate the transcription factor STAT1 were included as positive controls (Fig 2). Because intracellular bacterial luminescence provided a robust and sensitive assay for *C*. *burnetii* replication (Fig 1B), the *C*. *burnetii lux* strain was used to monitor the effect of individual gene knockdowns on bacterial replication. Silencing of genes encoding components of the JAK-STAT signal transduction pathway, *IFNγR1*, *IFNγR2*, *JAK1*, *JAK2* and *STAT1*, significantly increased *C*. *burnetii* replication in IFNγ-treated cells, which indicated that this screen was sensitive enough to identify potential restriction factor candidates (Fig 2). Among the IFNγ-induced effector genes, silencing of the gene encoding *Indoleamine 2*,*3-dioxygenase 1* (*IDO1*) resulted in the largest increase in *C*. *burnetii* replication in IFNγ-treated cells (Fig 2). These data implicate IDO1 as being a critical effector that mediates restriction of *C*. *burnetii* intracellular replication in IFNγ-treated cells.

### IDO1 is an IFNγ-induced effector that restricts *C*. *burnetii* replication

**Fig 3:**
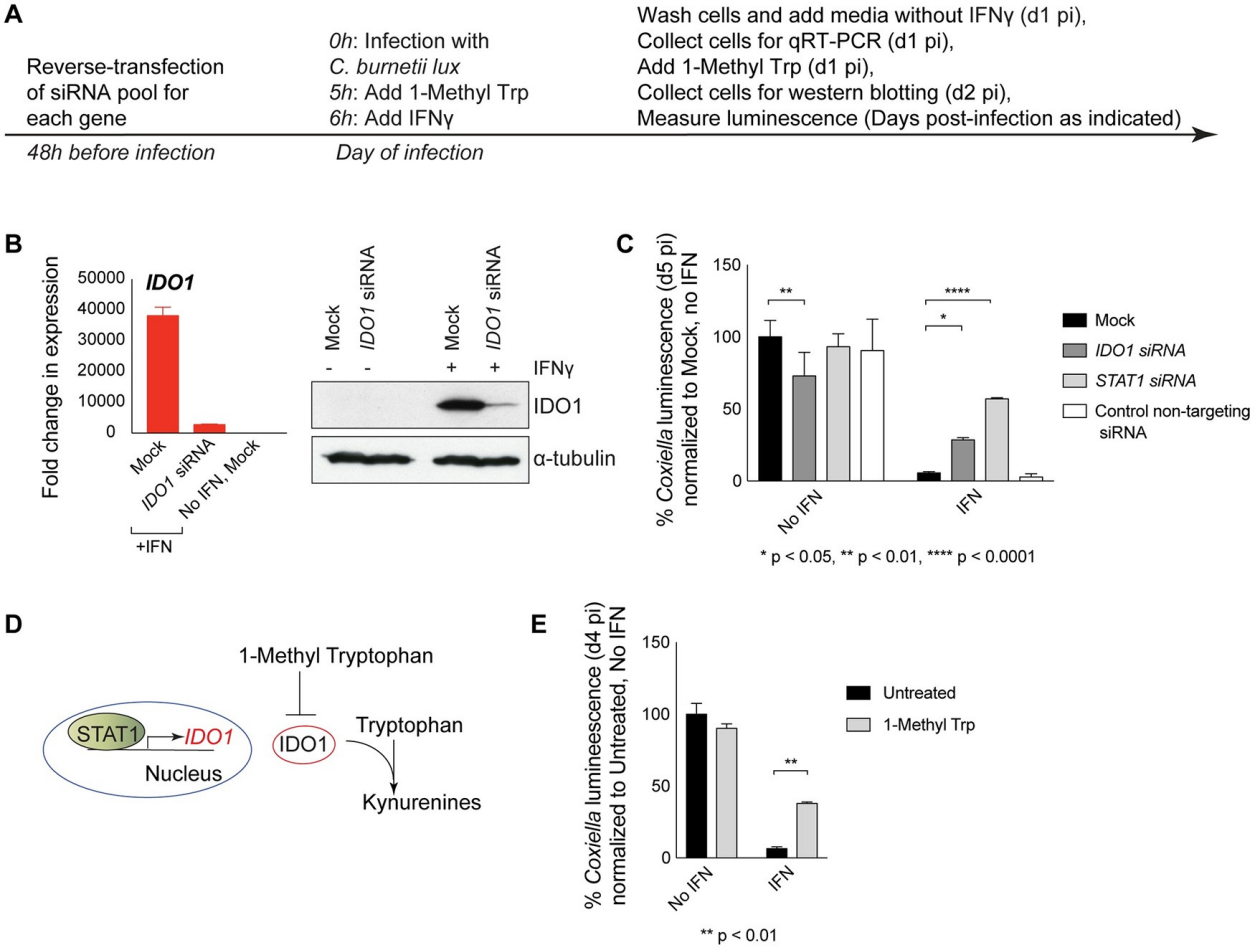
Indoleamine 2,3-dioxygenase 1 (IDO1) enzymatic activity is important for restricting *C*. *burnetii* replication. Experimental setup is laid out in A. Induction of IDO1 expression by IFNγ and knockdown efficiency of siRNA were determined by qRT-PCR and western blotting (B). Knocking-down *IDO1* by siRNA (C) or blocking the enzymatic activity of IDO1 by 1-Methyl tryptophan (E) allows replication of *C*. *burnetii lux* in IFNγ-treated cells but does not increase *C*. *burnetii* luminescence in untreated cells. Mechanism of action of IDO1 is depicted in D.

Experiments to validate that IDO1 expression inhibits *C*. *burnetii* replication were conducted in HeLa cells (Fig 3A). Immunoblot analysis and quantitative RT-PCR demonstrated robust induction of IDO1 in IFNγ-treated cells, and silencing of IDO1 expression by siRNA treatment (Fig 3B). As suggested in the initial screen, *IDO1* silencing significantly increased *C*. *burnetii* luminescence in IFNγ-treated cells (Fig 3C). In *STAT1*-silenced cells it was found that IFNγ treatment resulted in higher levels of *C*. *burnetii* luminescence, which indicates that there are additional mechanisms by which IFNγ stimulation of cells restricts intracellular replication of *C*. *burnetii* in the absence of IDO1 (Fig 3C). IDO1 is a cytosolic enzyme that catabolizes tryptophan, which will reduce cellular levels of this amino acid. This enzyme can be inhibited by 1-Methyl Trp, which is a competitive analogue of tryptophan (Fig 3D). The addition of 1-Methyl Trp enhanced *C*. *burnetii* luminescence in IFNγ-treated cells to a magnitude similar to that observed when *IDO1* was silenced (Fig 3E). Thus, the enzymatic activity of IDO1 is important for restriction of *C*. *burnetii* in host cells stimulated with IFNγ.

### IDO1 inhibits *C*. *burnetii* replication by depleting cellular pools of tryptophan

**Fig 4:**
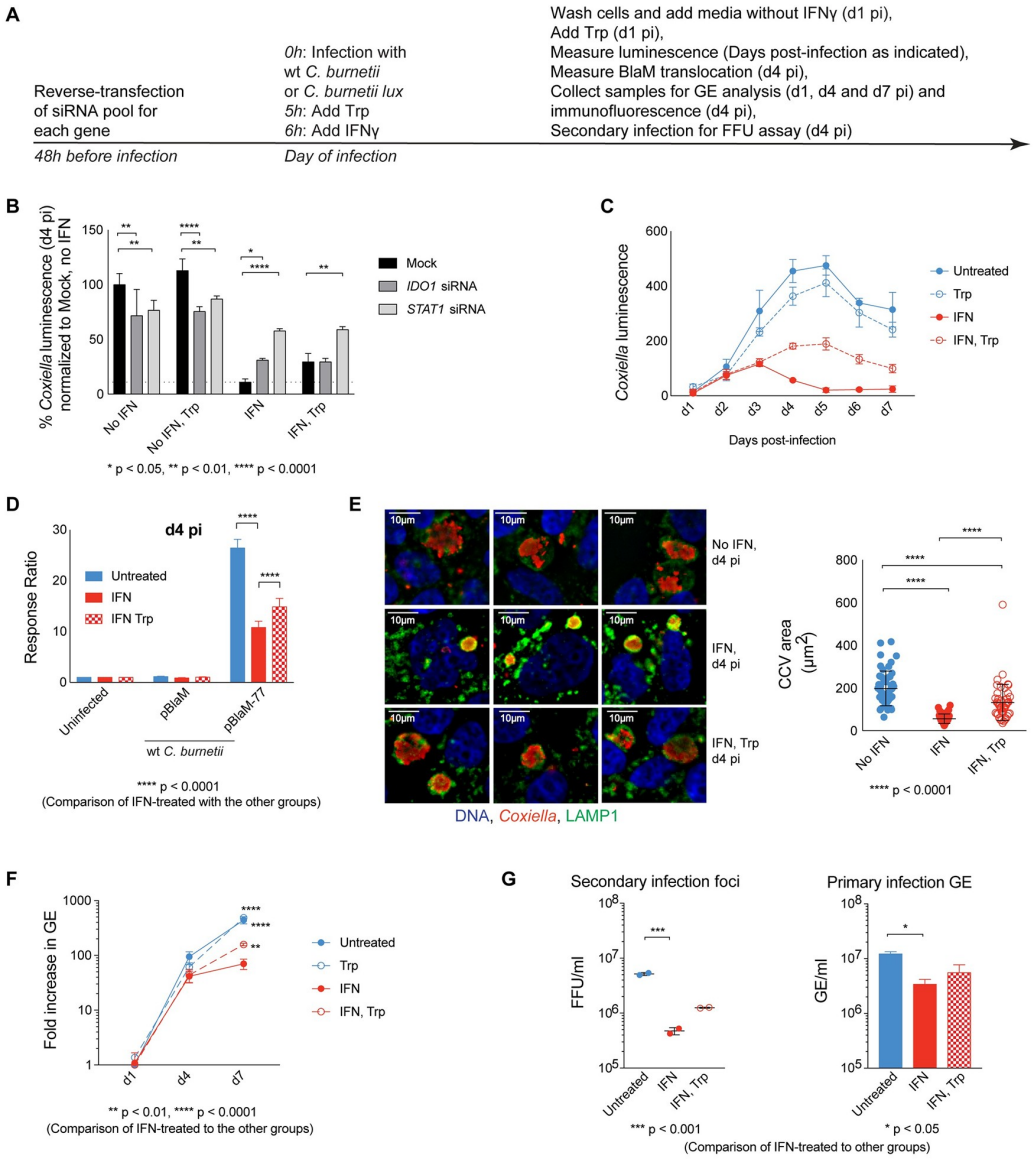
Tryptophan depletion is the mechanism of IDO1-mediated inhibition of *C*. *burnetii* replication. Experimental setup is laid out in A. Knocking down *IDO1* by siRNA (B) or supplementing the media with tryptophan (B and C) allows replication of *C*. *burnetii lux* in IFNγ-treated cells as measured by luminescence. HeLa cells were infected with wt *C*. *burnetii* pBlaM or pBlaM-77 and left untreated or treated with IFNγ in the presence or absence of additional tryptophan. On d4 pi, cells were loaded with the fluorescent substrate CCF4-AM and translocation of BlaM was assessed by the shift in the fluorescence emission from 535 to 460nm relative to that of uninfected (D). CCV were visualized by indirect immunofluorescence on d4 pi and average CCV sizes were quantified for about 40–50 CCVs per experimental condition (E). Fold increase in GE on d4 and d7 pi, compared to that of d1 pi were measured by qPCR (F). FFU/ml of bacteria derived from HeLa cells infected with *C*. *burnetii* in the presence or absence of IFNγ and Trp were determined by secondary infection of HeLa cells (G, left panel). As a control, GE was measured after primary infection (G, right panel).

Because *C*. *burnetii* is a tryptophan auxotroph it must acquire tryptophan from the host cell [25]. IDO1 expression could inhibit *C*. *burnetii* replication by depleting tryptophan in the cytosol or by generating kynurenines that have antimicrobial properties. To determine if tryptophan depletion was the primary mechanism by which IDO1 inhibits *C*. *burnetii* replication, exogenous tryptophan was added to the tissue culture medium to test whether this was sufficient to suppress IDO1-mediated growth restriction. A schematic showing the timing of tryptophan addition and collection of samples is shown in Fig 4A. Tryptophan supplementation increased *C*. *burnetii* luminescence in IFNγ-treated cells to a magnitude similar to that observed following *IDO1* knockdown (Fig 4B). Importantly, tryptophan supplementation did not enhance *C*. *burnetii* luminescence in *IDO1-* or *STAT1*-silenced cells. This indicates that growth restriction mediated by tryptophan depletion is dependent on STAT1 and IDO1. A decrease in *C*. *burnetii* luminescence was observed in the IFNγ-treated cells 3-days post-infection, consistent with a cessation of luciferase production by intracellular bacteria (Fig 4C). By contrast, *C*. *burnetii* luminescence values continued to increase over the 5-days of infection when the IFNγ-treated cells were supplemented with tryptophan, which indicates that the intracellular bacteria remained more metabolically active (Fig 4C). Similarly, tryptophan supplementation augmented the function of the type IV secretion system in IFNγ-treated cells as evident from the significant increase in the translocation of the effector protein BlaM-Cbu0077 (Fig 4D). Measurements of CCV area showed that IFNγ treatment resulted in smaller vacuoles containing *C*. *burnetii*, and this phenotype was suppressed by the addition of tryptophan (Fig 4E). To gain additional insight into the mechanism of IDO1-mediated growth restriction, a GE assay was used to measure *C*. *burnetii* genome expansion. Similar to the luminescence data, a decrease in *C*. *burnetii* GE values was observed in the IFNγ-treated cells compared to untreated cells at 4-days post-infection and tryptophan supplementation of cells treated with IFNγ resulted in a significant increase in *C*. *burnetii* GE values by d7 pi (Fig 4F). Lastly, the FFU assay showed that the addition of tryptophan increased *C*. *burnetii* viability in the IFNγ-treated cells (Fig 4G). Thus, IDO1 production interferes with *C*. *burnetii* intracellular replication and survival by depleting free tryptophan in the host cytosol and supplementing the culture medium with excess tryptophan suppresses IDO1-mediated restriction.

### IDO1-mediated tryptophan depletion results in a defect in CCV maintenance

**Fig 5:**
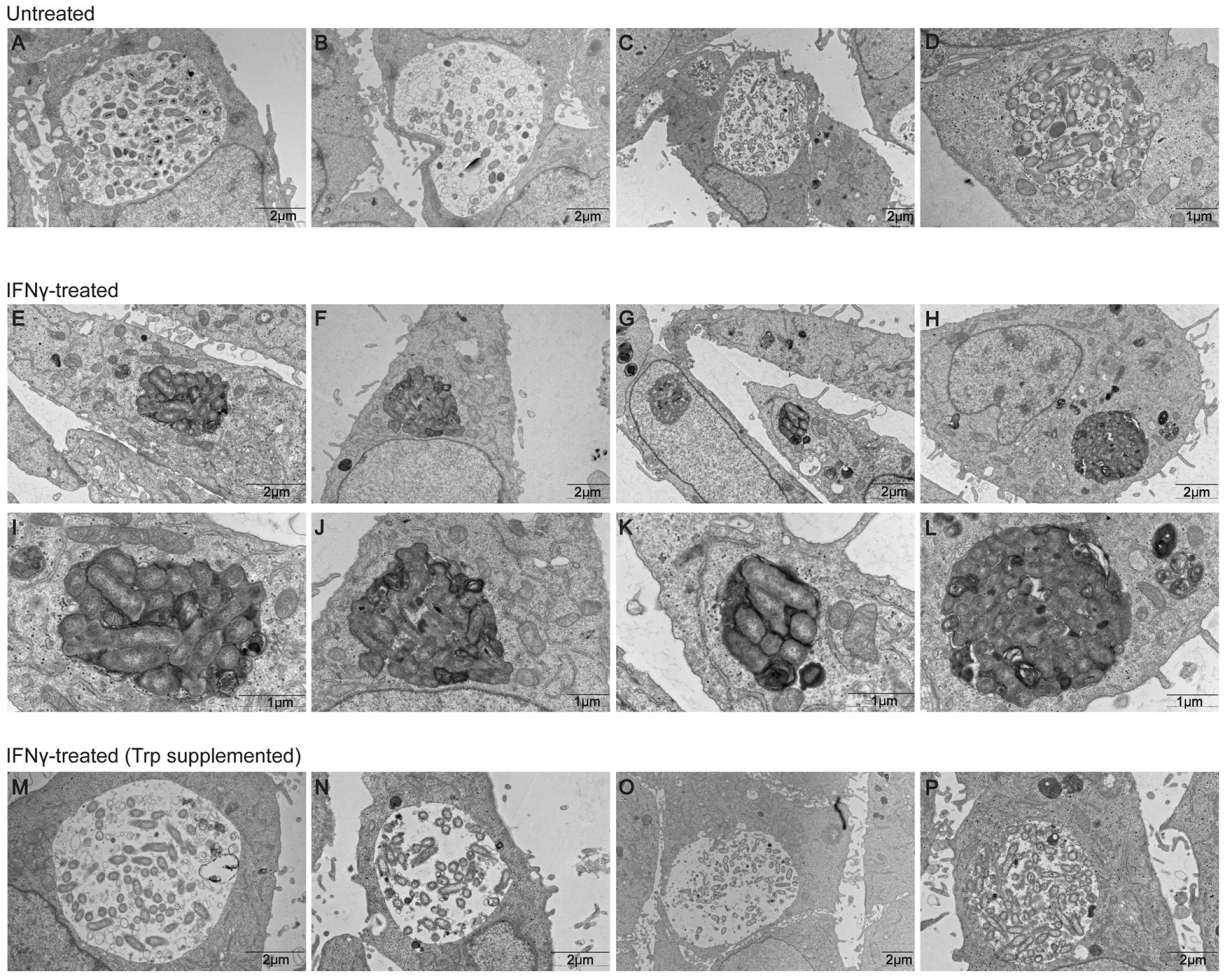
IDO1-mediated depletion of tryptophan interferes with the size and morphology of the CCV. *C*. *burnetii*-infected cells were left untreated or treated with IFNγ in the presence or absence of tryptophan as per the experimental layout in Fig 4A. Representative TEM images from untreated (A-D), IFNγ-treated (E-L) and tryptophan supplemented, IFNγ-treated (M-P) cells on d4 pi are presented here. I, J, K and L are higher magnification images of E, F, G and H respectively. Scale bars are denoted at the bottom right of each image.

Transmission electron microscopy (TEM) was used to examine whether IFNγ stimulation had any detectible impact on the morphology of the CCV. Several representative images show that CCVs in untreated cells were spacious, which means the individual *C*. *burnetii* were dispersed randomly throughout the lumen of the vacuole and were not typically in close contact with each other. Also, numerous vesicles were observed within the lumen of CCVs, which is likely due to robust fusion of autophagosomes with the CCV and formation of internal vesicles by a functional multivesicular body (MVB) pathway (Fig 5). By contrast, CCVs in IFNγ-treated cells were constricted, showed enhanced osmium tetroxide staining, had fewer intraluminal vesicles, and the bacteria inside the vacuole were tightly packed and many showed signs of damage resulting in cellular swelling (Fig 5). Importantly, the membrane of the CCV appeared to be intact and *C*. *burnetii* were not detected in the cytosol. The CCVs in the IFNγ-treated cells supplemented with tryptophan were similar in appearance to the CCVs in the untreated cells. Thus, tryptophan depletion resulting from IDO1 production interferes with the ability of *C*. *burnetii* to maintain a spacious CCV that supports intracellular replication.

### IDO1 expression is sufficient to restrict the intracellular replication of *C*. *burnetii*

**Fig 6:**
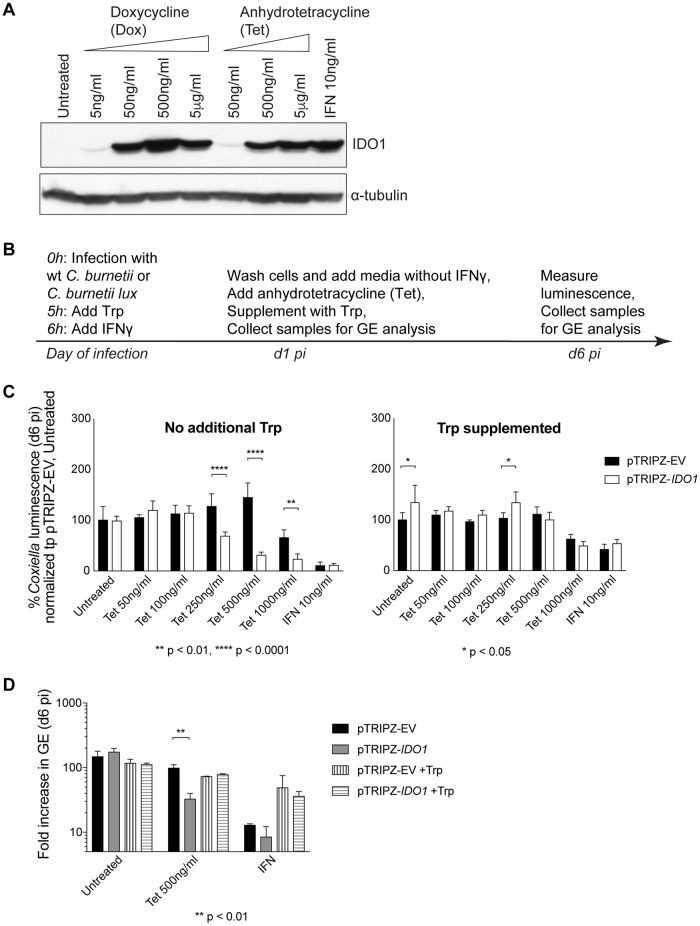
IDO1 expression is sufficient to restrict the intracellular replication of *C*. *burnetii*. HeLa cells were stably transduced with an empty pTRIPZ lentiviral vector (pTRIPZ-EV) or one where *IDO1* was cloned under the control of tetracycline-inducible promoter (pTRIPZ-*IDO1*). Uninfected pTRIPZ-*IDO1* cells were treated with different concentrations of doxycycline (referred to as Dox) or anhydrotetracycline (referred to as Tet) for 3 days or IFNγ for 4 days and lysates tested for IDO1 expression by western blotting (A). Panel B lays out the experimental setup for results shown in C and D. pTRIPZ-EV or pTRIPZ-*IDO1* cells were infected with *C*. *burnetii lux* (C) or wt *C*. *burnetii* (D) and treated with IFNγ or Tet at indicated time points (B). Luminescence was measured in the absence (left panel) or presence of supplemented tryptophan (right panel) and normalized to that of untreated, pTRIPZ-EV cells (C). Media was supplemented with tryptophan on the day of infection as well as on d1 pi for IFNγ-treated cells and on d1 pi for Tet-treated cells (C, right panel). *C*. *burnetii* GE measured on d6 pi was normalized to that of d1 pi and presented as fold increase (D).

Although IDO1 was important for restriction of *C*. *burnetii* replication in cells stimulated with IFNγ, it was unclear whether IDO1 expression would restrict *C*. *burnetii* replication in the absence of other IFNγ-induced genes. To address this question, a HeLa cell line that produces IDO1 under the control of a tetracycline-inducible promoter was created. Immunoblot analysis showed that the IDO1 protein was not produced by unstimulated cells but when cells were stimulated with a tetracycline inducer (doxycycline or anhydrotetracycline) or treated with IFNγ, there was a concentration-dependent increase in IDO1 protein levels (Fig 6A). The IDO1-inducible cell line was infected with *C*. *burnetii lux* and IDO1 expression was stimulated by either anhydrotetracycline (referred to as Tet) induction or by stimulation with IFNγ as shown in the schematic (Fig 6B). Anhydrotetracycline induction of IDO1 significantly inhibited *C*. *burnetii* replication in the HeLa pTRIPZ-*IDO1* cells, but not in the vector control HeLa pTRIPZ-EV cells (Fig 6C, left panel). The addition of tryptophan to the culture medium restored *C*. *burnetii* replication in HeLa pTRIPZ-*IDO1* cells that had been induced to produce IDO1 (Fig 6C, right panel). The GE assay confirmed data from the luminescence assay and showed that induction of IDO1 production in the HeLa pTRIPZ-*IDO1* cells was sufficient to restrict *C*. *burnetii* replication by a mechanism that could be suppressed by the addition of tryptophan to the culture medium (Fig 6D). These data indicate that host cell expression of IDO1 will restrict *C*. *burnetii* replication in the absence of IFNγ signaling, which validates that depletion of tryptophan in the host cell cytosol is sufficient to disrupt the ability of *C*. *burnetii* to replicate in the lysosome-derived CCV.

### IDO1 participates in IFNγ-mediated restriction of *C*. *burnetii* replication in human macrophages

**Fig 7:**
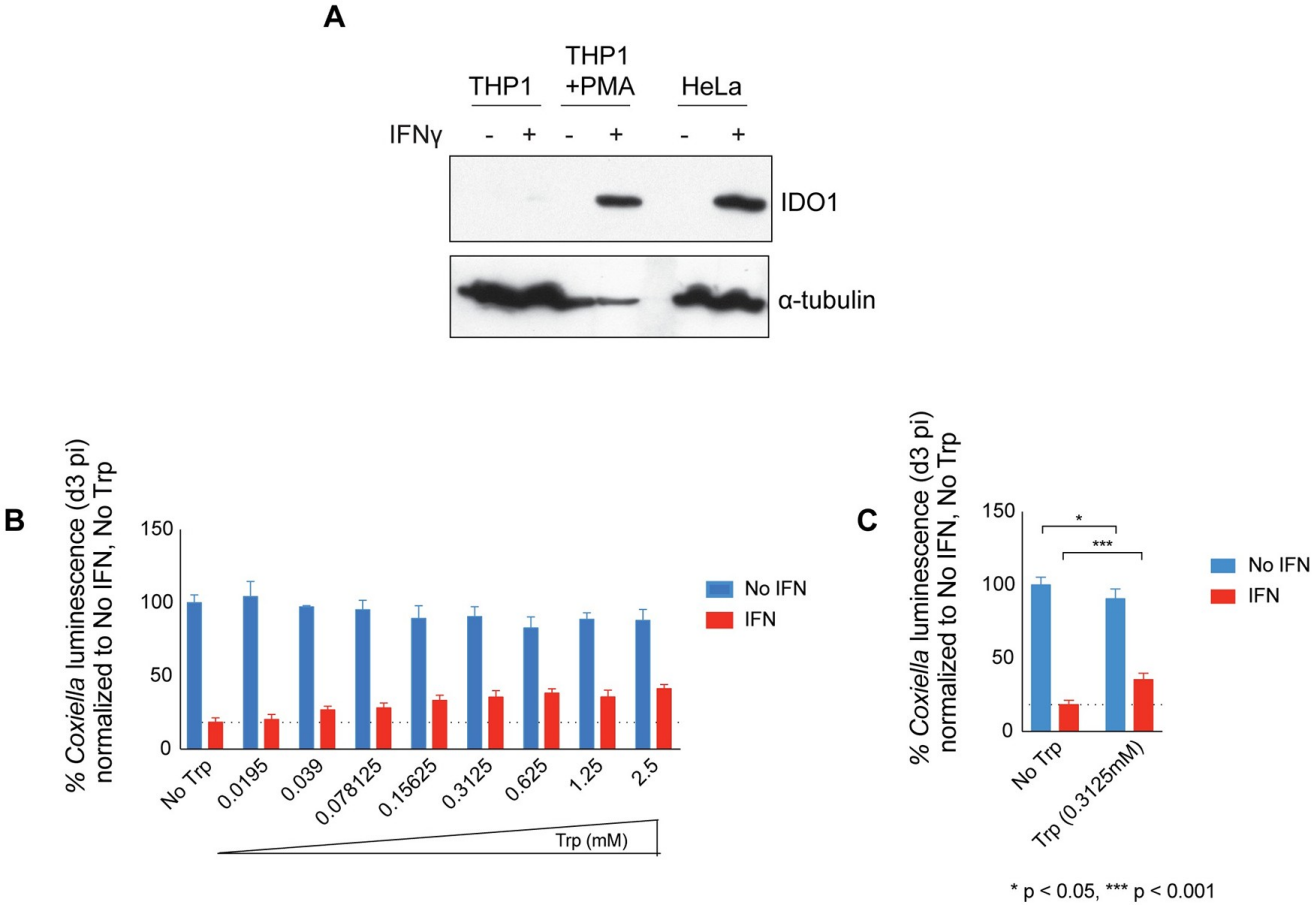
IDO1 contributes to IFNγ-mediated growth restriction of *C*. *burnetii* in human macrophages. Undifferentiated or PMA-differentiated THP1 cells and HeLa cells were left untreated or treated with IFNγ. On d2 post IFNγ-treatment, cell lysates were collected and IDO1 protein levels observed by immunoblotting (A). PMA-differentiated THP1 macrophages were infected with *C*. *burnetii lux* and treated with IFNγ in the presence or absence of additional Trp at the concentrations indicated. Luminescence values on d3 pi were normalized to that of untreated, no-Trp supplemented cells (B). Data specific for Trp concentration 0.3125mM from Fig 7B is represented in Fig 7C. Dotted lines indicate the normalized luminescence values for IFNγ-treated, no-Trp supplemented cells based on which statistical significance was calculated (B and C).

The THP1 cell line was used to determine whether IDO1-mediated restriction of *C*. *burnetii* replication is a conserved IFNγ-activated pathway that is operational in human-derived macrophage-like cells. Immunoblot analysis was used to evaluate IDO1 production in undifferentiated THP1 cells and THP1 cells that were differentiated into macrophage-like cells by treatment with PMA. Similar to control HeLa cells, differentiated THP1 cells produced IDO1 protein upon IFNγ stimulation (Fig 7A). Differentiated THP1 cells treated with IFNγ restricted *C*. *burnetii* replication as determined by measuring bacterial luminescence (Fig 7B). The culture medium was supplemented with increasing amounts of tryptophan to determine if IDO1-mediated tryptophan depletion has a measurable effect on restriction of *C*. *burnetii* replication in differentiated THP1 cells treated with IFNγ. A dose-dependent increase in *C*. *burnetii* luminescence was observed upon the addition of tryptophan (Fig 7B). A tryptophan concentration of 0.3125 mM resulted in significantly higher levels of *C*. *burnetii* luminescence (Fig 7C). Thus, IDO1 is induced and participates in the restriction of *C*. *burnetii* replication in macrophages.

## Discussion

Activation of cell-intrinsic defense by the cytokine IFNγ enables mammals to combat a large number of microbial pathogens that are able to survive and replicate in host cells. In this study, *C*. *burnetii* was used as a model pathogen to advance our understanding of how IFNγ-induced responses enable host cells to defend themselves against pathogens that have evolved the ability to replicate in lysosomes, which are catabolic and hostile organelles for most microbes. The data revealed that IFNγ, even at relatively low doses, stimulated a response that efficiently restricted *C*. *burnetii* replication in HeLa cells, which indicates that non-phagocytic cells are also equipped with cell-intrinsic antimicrobial mechanisms that can limit replication of this pathogen. The IFNγ-induced enzyme IDO1 was found to be important for restriction of *C*. *burnetii* replication. IDO1 limits the amount of tryptophan available for *C*. *burnetii*, which is a tryptophan auxotroph so must acquire this essential nutrient from the host. Data presented here indicate that IDO1-mediated depletion of tryptophan stalls *C*. *burnetii* infection by inhibiting bacterial metabolism and secretion of effector proteins by the Dot/Icm system. This would explain the impact of IDO1 production on the size, morphology and maintenance of the CCV, which is dependent on Dot/Icm function.

Importantly, IDO1 is an enzyme that remains localized in the host cell cytosol, whereas, *C*. *burnetii* replicates inside a membrane-bound vacuole. Given that the lysosome-derived CCV is an acidified organelle and retains the ability to hydrolyze proteins, these data suggest that the pool of tryptophan generated by lysosomal degradation of proteins inside the CCV is not sufficient to maintain the nutritional requirements for *C*. *burnetii* metabolism. Thus, this organism must have the ability to access metabolites such as tryptophan that are in the cytosol. In addition to tryptophan, *C*. *burnetii* depends on the host for several other key amino acids, which include arginine, cysteine, histidine, leucine, lysine, phenylalanine, proline, tyrosine, threonine, and valine [25]. An important question for future studies will be to determine which of these essential amino acids can be generated in sufficient quantities by hydrolysis of proteins in the vacuole lumen, and which amino acids must be imported into the vacuole either through the subversion of host transporters or through bacterial transporters that are delivered into the CCV membrane.

Phylogenetic analysis of the *C*. *burnetii* genome indicates that genome reduction, pseudogenization of genes occurred as *Coxiella* evolved and adapted from tick-associated lifestyle to infect mammalian hosts [32–36]. *C*. *burnetii* tryptophan synthesis genes, in particular, are related to the genes in *Simkania negevensis*, which is in the phylum Chlamydiae and have been suggested to be acquired from Chlamydial ancestors through horizontal transfer [36,37]. A closer analysis of the *C*. *burnetii trp* genes provides insight into why this pathogen is unable to synthesize tryptophan [32,38]. Multiple frameshift mutations in the *trpDG* genes (CBU1153) renders the biosynthetic pathway incapable of utilizing chorismate as a precursor for tryptophan synthesis [32]. There is also a fusion of the genes encoding phosphoribosyl anthranilate isomerase (*trpF*) and tryptophan synthase (*trpB*) [32]. Whether this fusion protein (CBU1155) retains enzymatic function is unknown. Regardless, data here indicate that IDO1-mediated depletion of tryptophan restricts *C*. *burnetii* replication, which means that *C*. *burnetii* is incapable of utilizing secondary metabolites in the tryptophan synthesis pathway or that these substrates are not sufficiently available.

*Chlamydia*, *Leishmania* and *Toxoplasma spp*. are also tryptophan auxotrophs and susceptible to IDO1-mediated growth restriction in human cells [39–42]. Similar to what was shown here for *C*. *burnetii*, the inclusion containing *Chlamydia trachomatis* becomes contrasted and *Chlamydia* exhibit enlarged, atypical morphology during IDO1-mediated tryptophan depletion [43]. Intracellular replication of *Mycobacterium tuberculosis* (Mtb) *and Listeria monocytogenes*, which are not tryptophan auxotrophs, can also be inhibited by IDO1 activity. For Mtb, IDO1-mediated tryptophan depletion is detrimental to mutants defective in tryptophan biosynthesis [44]. In the case of *Listeria*, tryptophan catabolites such as kynurenines (Kyn) and 3-hydroxy kynurenines (3HK) have been shown have antibacterial effects [45]. It is unlikely that kynurenines have a negative impact on *C*. *burnetii* replication because supplementing culture medium with excess tryptophan completely negated the contribution of IDO1 to restricting *C*. *burnetii* replication. Additionally, presence of excess tryptophan in the axenic medium used to culture *C*. *burnetii* has been shown to maximize log-phase growth and bacterial plating efficiency [8,46]. Thus, tryptophan is essential for *C*. *burnetii* multiplication in both cell-free and intracellular environments.

Several IFNγ-induced effectors did not appear to be necessary for restriction of *C*. *burnetii* replication based on this screen. For instance, silencing of the chloride, glucose and organic compound transporter genes (*CLIC2*, *SLC2A3*, *SLC16A1* and *SLC22A2*), or the genes involved in phospholipid and cholesterol transport (*PLSCR1*, *APOL1* and *APOL2*), did not increase *C*. *burnetii* replication. Likewise for the genes encoding the gp91-phox subunit of the NADPH oxidase (*CYBB*) or nitric oxide synthase (*NOS2*). This may be explained by the finding that intracellular bacteria such as *Salmonella* do not experience significant redox stress in epithelial cells compared to macrophages [47]. In addition, *C*. *burnetii* is believed to encode acid phosphatases, superoxide dismutases and peroxiredoxin that neutralize the toxic effect of oxidative radicals [48–52]. Induction of apoptotic cell death pathways is another mechanism by which IFNγ-treated cells can restrict the replication of intracellular pathogens. Bacterial effector proteins delivered into host cell by the *C*. *burnetii* T4SS, however, can block intrinsic and extrinsic apoptotic pathways in infected epithelial cells [53,54], which may be why silencing of the TNF receptor (*TNFRSF1A*) did not have an effect on *C*. *burnetii* growth restriction. Lastly, it is well appreciated that interferon-inducible guanylate binding proteins and the immunity-related GTPase M (GBP1, GBP2, GBP5 and IRGM) target pathogen-containing compartments and cytosolic bacteria to promote their destruction by cell-autonomous mechanisms (reviewed in [55]). These GTPases were not essential for inhibiting *C*. *burnetii* replication. Because *C*. *burnetii*-containing phagosomes are transported along the default endocytic pathway to the lysosomes, these GTPases may not recognize the CCV as a modified phagosome that has evaded maturation and should be targeted for lysosomal fusion and degradation. It remains possible that some of these IFNγ-induced proteins contribute to *C*. *burnetii* growth restriction, but because they may have functions that are redundant, there is no enhancement of *C*. *burnetii* replication when only one factor is silenced in IFNγ-treated cells.

Data presented here demonstrate that ectopic production of IDO1 is sufficient to suppress *C*. *burnetii* replication in the absence of IFNγ signaling. IDO1 activity can limit the replication of intracellular pathogens to prevent dissemination, however, is typically not sufficient to kill intracellular microbes and without the participation of other cell autonomous defense pathways, can lead to persistent infection of infected tissues [56,57]. It is likely that other IFNγ-dependent mechanisms participate in killing *C*. *burnetii* once IDO1 renders the bacteria metabolically incapable of interfering with host cell functions. Consistent with IFNγ-activation leading to nutritional depletion, knockdown of *SLC11A1*, the gene encoding the metal transporter NRAMP1 (Natural resistance-associated macrophage protein 1) that restricts iron and manganese levels in phagosomes, led to a small, but significant increase in *C*. *burnetii* replication. These data indicate that host modulation of metal concentrations in the CCV could contribute to restriction of intracellular *C*. *burnetii*. Thus, future studies will be aimed at identifying additional IFNγ-dependent mechanisms that act synergistically to combat intracellular *C*. *burnetii* replication using cells that are deficient in IDO1.

## Materials and methods

### Cell lines

HeLa 229 cells (ATCC) and THP1 (ATCC) were used in experiments. HeLa cells were cultured and maintained in Dulbecco’s Modified Eagle Medium (DMEM, Gibco Cat. 11965–118) supplemented with 10% heat-inactivated fetal bovine serum (FBS). THP1 cells were cultured in Roswell Park Memorial Institute 1640 Medium with ATCC modification (ThermoFisher Scientific, Cat. A1049101) supplemented with 10% heat-inactivated FBS. All cells were maintained at 37°C with 5% CO_2_. During the length of infection experiments, HeLa cells were maintained with 5% FBS whereas THP1 cells were maintained with 10% FBS.

### *Coxiella burnetii* strains

Wild-type *Coxiella burnetii* Nine Mile phase II (RSA439) was cultured in ACCM-2 media and used for all experiments [58,59]. A NMII strain that constitutively expresses luminescence was generated and provided by Shawna C. Reed (Table 1). *C*. *burnetii* strains were grown for 6 days in ACCM-2 at 37°C, 2.5% O_2_ and 5% CO_2_ with appropriate antibiotic selection (375μg/ml kanamycin for Kan^r^ strains, 3μg/ml chloramphenicol for Cm^r^ strains) as described [58,59]. Bacterial cultures were centrifuged at 4000 rpm, 4°C for 15 mins and pellets re-suspended in half the volume with DMEM containing 5% FBS. Bacteria were sonicated for 10’ prior to infection. *C*. *burnetii* genome equivalents were measured by quantitative PCR as described previously [27].

**Table 1:** *C*. *burnetii* strains used in this study.

Strain	Antibiotic resistance	Genotype/Generation	Reference
Wild-type (wt) Nine Mile phase II (NMII) RSA439			
*C*. *burnetii lux* (generated and gifted by Shawna C. Reed, Roy Laboratory, Yale University)	Kan^r^	Single copy attTN7::KAN-P311-*luxCDABE*-TT genomic insertion via plasmid with *cbu0311* promoter and a terminator cloned into pMiniTn7T-KAN-*luxCDABE* (gift from Paul A. Beare, NIAID, NIH, Montana, USA)	[60]
*icmL*::Tn *C*. *burnetii*	Kan^r^	Transposon insertion in *icmL*.*1/dotI* (*cbu1629*)	[3]

### Reagents

Black 96-well plates with clear bottom were purchased from Corning costar (Cat. 3904). Dharmafect (T-2001), 5X siRNA buffer (Cat. B-002000-UB-100) and siGENOME SMARTpool siRNAs as listed in Table 2, were purchased from Dharmacon. Recombinant human IFNγ was obtained from Biolegend (Cat. 570206). L-tryptophan (Trp) and 1-Methyl L-tryptophan (1-Methyl Trp) were obtained from Sigma-Aldrich (Cat. T0254, 447439). Trp was re-suspended in Tissue Culture grade water and used at 0.3125mM unless otherwise indicated. 1-Methyl Trp was resuspended in 0.1N NaOH and used at 0.2mM. Phenol-red free DMEM and probenecid were obtained from Thermo Fisher Scientific (Cat. 21063029 and P36400). LIVEBLAzer^™^-FRET B/G loading kit which includes the fluorescent substrate CCF4-AM was purchased from Invitrogen (Cat. K1095).

**Table 2:** SMARTpool siRNA from Dharmacon.

Pool Catalog Number	Gene Symbol	GENE ID	Gene Accession	GI Number	Gene Description
M-011057-00	IFNGR1	3459	NM_000416	4557879	interferon gamma receptor 1
M-012713-00	IFNGR2	3460	NM_005534	47419933	interferon gamma receptor 2
M-003146-02	JAK2	3717	NM_004972	13325062	Janus kinase 2
M-003145-02	JAK1	3716	NM_002227	102469033	Janus kinase 1
M-011704-01	IRF1	3659	NM_002198	4504720	interferon regulatory factor 1
M-020858-02	IRF9	10379	NM_006084	82734235	interferon regulatory factor 9
M-003543-01	STAT1	6772	NM_007315	21536299	signal transducer and activator of transcription 1
M-003544-02	STAT3	6774	NM_213662	47458819	signal transducer and activator of transcription 3
M-006423-03	CEBPB	1051	NM_005194	28872795	CCAAT enhancer binding protein beta
M-009240-01	NOS2	4843	NM_000625	24041028	nitric oxide synthase 2
M-011021-01	CYBB	1536	NM_000397	163854302	cytochrome b-245 beta chain
M-005153-02	GBP1	2633	NM_002053	4503938	guanylate binding protein 1
M-011867-00	GBP2	2634	NM_004120	38327557	guanylate binding protein 2
M-028450-01	IRGM	345611	XM_001127260	113416797	immunity related GTPase M
M-014116-01	IFITM3	10410	NM_021034	148612841	interferon induced transmembrane protein 3
M-019469-01	STX11	8676	NM_003764	33667037	syntaxin 11
M-008317-00	RAB20	55647	NM_017817	8923400	RAB20, member RAS oncogene family
M-028161-01	RAB43	339122	NM_198490	50234888	RAB43, member RAS oncogene family
M-010337-01	IDO1	3620	NM_002164	156071492	indoleamine 2,3-dioxygenase 1
M-019310-01	IDO2	169355	NM_194294	148539553	indoleamine 2,3-dioxygenase 2
M-013432-01	APOL6	80830	NM_030641	87162462	apolipoprotein L6
M-017402-02	APOL1	8542	NM_003661	21735613	apolipoprotein L1
M-007380-02	SLC11A1	6556	NM_000578	109255240	solute carrier family 11 member 1
M-009553-00	RAB36	9609	NM_004914	31795534	RAB36, member RAS oncogene family
M-011002-01	C4A	720	NM_007293	67190747	complement C4A
M-005197-00	TNFRSF1A	7132	NM_001065	23312372	TNF receptor superfamily member 1A
M-011511-04	SOCS1	8651	NM_003745	4507232	suppressor of cytokine signaling 1
M-003502-01	ICAM1	3383	NM_000201	4557877	intercellular adhesion molecule 1
M-012982-02	CLIC2	1193	NM_001289	66346732	chloride intracellular channel 2
M-003729-03	PLSCR1	5359	NM_021105	10863876	phospholipid scramblase 1
M-008322-01	WARS	7453	NM_004184	7710155	tryptophanyl-tRNA synthetase
M-004202-01	NMI	9111	NM_004688	4758813	N-myc and STAT interactor
M-005844-01	CTSS	1520	NM_004079	23110961	cathepsin S
M-007516-01	SLC2A3	6515	NM_006931	5902089	solute carrier family 2 member 3
M-007402-02	SLC16A1	6566	NM_003051	115583684	solute carrier family 16 member 1
M-007453-00	SLC22A2	6582	NM_003058	23510411	solute carrier family 22 member 2
M-007143-01	DTX3L	151636	NM_138287	31377615	deltex E3 ubiquitin ligase 3L
M-018178-00	GBP5	115362	NM_052942	31377630	guanylate binding protein 5
M-007831-01	CCL2	6347	NM_002982	56119169	C-C motif chemokine ligand 2
M-006022-01	PSMB8	5696	NM_148919	73747874	proteasome subunit beta 8
M-011699-01	IRF8	3394	NM_002163	55953136	interferon regulatory factor 8
D-001810-10	ON-TARGETplus Non-targeting Control				
L-003543-00-005 (ON-TARGETplus SMARTpool siRNA)	STAT1	6772			signal transducer and activator of transcription 1

### siRNA screen and luminescence measurement

siRNAs were re-suspended in 1X siRNA buffer as 10μM or 2μM stocks and stored at -20°C until use. 48h before infection, HeLa cells (10^4^ cells/well) were reverse-transfected with 25 or 50nM siRNA using Dharmafect (0.2μl/well) in 96-well black, clear bottom plates. As controls, cells were transfected with transfection reagent alone (Mock) or control non-targeting siRNA. HeLa cells were infected with *C*. *burnetii lux* at MOI 100 and 6h later, IFNγ was added. Cells were washed and replenished with fresh media without IFNγ on d1 pi and luminescence values measured on specific days post-infection as indicated. Peak *C*. *burnetii* luminescence values measured from untreated cells (d4 pi or as indicated) were normalized to 100%. In the experiments performed with Trp and 1-Methyl Trp, *C*. *burnetii*- infected cells were supplemented with Trp (0.3125mM) or treated with 1-Methyl Trp (0.2mM) 1h prior to treatment with IFNγ. Trp or 1-Methyl Trp, but not IFNγ, were added back to the media after washing the cells on d1 pi. Bacterial luminescence was measured using TECAN infinite M1000 plate reader on indicated days post-infection.

### Assessing bacterial luminescence in THP1 cells

THP1 cells were plated at 75000/well in 96-well plates and differentiated with 124ng/ml phorbol 12-myristate 13-acetate (PMA) overnight or left undifferentiated. Cells were infected with MOI 25 and 6h later, treated with IFNγ 100ng/ml in the presence or absence of additional tryptophan (concentrations as indicated). On d1 pi, cells were washed and replenished with IFNγ-free media and supplemented with tryptophan. Bacterial luminescence was measured using TECAN infinite M1000 plate reader on indicated days post-infection.

### BlaM translocation assay

Effector translocation through the type IV secretion system was assessed by measuring the translocation of β-lactamase (BlaM)- effector fusion protein using a FRET-based assay, as previously described [2]. HeLa cells were plated at 2*10^4^ cells/w in 96w black, clear bottom plates 24h prior to infection. Cells were infected with wt or *icmL*::Tn *C*. *burnetii* expressing BlaM alone or BlaM-77 at MOI 500 as listed in Table 3. Cells were treated with IFNγ 6h later, in the presence or absence of additional tryptophan. On d1 pi, cells were washed and replenished with IFNγ-free media and supplemented with tryptophan where indicated. On d2 or d4 pi, culture medium was replaced with phenol red-free DMEM containing HEPES. Cells were loaded with the fluorescent substrate CCF4/AM using the LIVEBLAzer^™^-FRET B/G loading kit and probenecid and incubated in dark at RT for 2h. The ratio of signal at 460 and 535nm (blue:green) was measured using the TECAN M1000 plate reader and the response ratio was calculated by normalizing the blue: green ratio of infected cells to that of uninfected control.

**Table 3:** Plasmids used in this study.

Plasmid and genotype	Notes	Antibiotic resistance	Reference/ Source
pBlaM (pJB-CAT-BlaM)	*C*. *burnetii* expression vector for β-lactamase (BlaM) fusion proteins (*cbu0069* promoter)	Cm^r^	[3]
pBlaM-77 (pJB-CAT-BlaM::*cbu0077*)	*C*. *burnetii* expression vector for BlaM-Cbu0077 fusion protein	Cm^r^	[3]
pTRIPZ-EV	Created by annealing the oligos YO-0903 and YO-0904 into the pTRIPZ Inducible Lentiviral shRNA vector (Dharmacon-Horizon Discovery) at Age-I/MluI sites	Amp^r^	Gift from Dr. Brett Lindenbach, Yale University
pTRIPZ-*IDO1*	Cloned human *IDO1* gene at EcorI site using ligase-independent cloning	Amp^r^	This study
pVSV-G	Lentiviral envelope plasmid	Amp^r^	[61]
psPAX2	Lentiviral packaging plasmid	Amp^r^	[62]

### Measurement of bacterial genome equivalents (GE)

5*10^4^ cells per well were plated in 24-well plates, one day prior to infection. Cells were infected wt *C*. *burnetii* NMII at MOI 100. Cells were left untreated or treated with IFNγ in the presence or absence of Trp at 6h pi. Infected cells were washed on d1 pi and replenished with IFNγ-free fresh media in the presence or absence of additional Trp. Supernatants and cells lysed with distilled water were combined and collected on d1, d4 and d7 pi. Genomic DNA was extracted using Illustra bacterial genomicPrep mini spin kit (Cat no. 28904259, GE) and quantified by qPCR using primers for *C*. *burnetii dotA* gene (Table 4).

**Table 4:** Primers and oligos used in this study.

Primer/ Oligo	Sequence	Purpose
IDO1 F	*gcccttcaagtgtttcaccaa*	qRT-PCR
IDO1 R	*gacaaatatatgcgaagaacactgaaaa*	qRT-PCR
GAPDH F	*agccacatcgctcagacac*	qRT-PCR
GAPDH R	*gcccaatacgaccaaatcc*	qRT-PCR
pTRIPZ-*IDO1* F	*acggcgcgccatcgatttaaatcctgcaggaattcatggcacacgctatggaaaac*	Cloning *IDO1* in pTRIPZ vector
pTRIPZ-*IDO1* R	*gcgcggaggccacgcgctcgagacgcgtgaattcttaaccttccttcaaaagggatttct*	Cloning *IDO1* in pTRIPZ vector
dotA F	*gcgcaatacgctcaatcaca*	qPCR for *C*. *burnetii* GE
dotA R	*ccatggccccaattctctt*	qPCR for *C*. *burnetii* GE
YO-0903	*ccggtgtacacggcgcgccatcgatttaaatcctgcaggaattcacgcgtctcgag*	Modifying pTRIPZ
YO-0904	*gcgcctcgagacgcgtgaattcctgcaggatttaaatcgatggcgcgccgtgtaca*	Modifying pTRIPZ

### Indirect immunofluorescence

2.5 or 5*10^4^ cells per well were plated in 24-well plates with poly L-lysine coated coverslips, one day prior to infection. Cells were infected at MOI 100, left untreated or treated with IFNγ and additional Trp as indicated. On d1 pi, cells were washed and replenished with IFNγ-free media in the presence or absence of additional Trp. Cells were fixed on d4 pi with 4% para formaldehyde (PFA) for 20 mins at RT. Coverslips were washed at least 6x times with 1x PBS, permeabilized and blocked with 0.2% saponin, 0.5% BSA and 1% (v/v) heat-inactivated FBS in PBS. Coverslips were stained with primary antibodies- rabbit anti-*C*. *burnetii* [3], mouse anti-LAMP1 (Source: H4A3 Development Studies Hybridoma bank at the University of Iowa) and DAPI (4,6-diamidino-2-phenylindole, Cat no. D9542 from Sigma-Aldrich) at 1:10,000, 1:500 and 1:10,000 dilutions respectively, as previously described [63]. Secondary antibodies goat anti-Rabbit IgG, Alexa Fluor 568 (Life Technologies, A11036) and goat anti-Mouse IgG, Alexa Fluor 488 (Life Technologies, A11029) were used at 1:2000. Stained coverslips were mounted on glass slides using Prolong gold antifade reagent (Life Technologies). Coverslips were visualized with a Photometrics CoolSNAP EZ 20 Mhz digital monochrome camera connected to Nikon Eclipse TE2000-S inverted microscope using Nikon Plan Apo60x objective/ 1.4 numerical aperture. Images were acquired using SlideBook software 6.0 (Intelligent Imaging Innovations), saved as tiff files and resized and labeled using Adobe Illustrator.

### FFU assay

To measure the infectivity of *C*. *burnetii* in infected cells, Foci Forming Unit (FFU) assay was used. HeLa 229 cells were plated in 6-well plates at 200,000 cells/w one day prior to infection. Cells were infected with wt *C*. *burnetii* at MOI 100, left untreated or treated with IFNγ and additional Trp as indicated. On d1 pi, cells were washed and replenished with IFNγ-free media in the presence or absence of additional Trp. Similar to GE assay, supernatants and lysates from infected cells were combined and collected on d4 pi and sonicated for 10’. This culture was serially diluted either in water (to assess primary infection GE by qPCR) or in DMEM containing 5% FBS to infect HeLa cells (plated at 2*10^4^ cells/well in 96-well plate on the previous day) for a second round of infection. Infected cells in 96-well plates were subsequently washed on d4 pi, fixed with 4% PFA for 20’ at RT, permeabilized with 0.5% Triton-X in PBS for 5’ and stained with rabbit anti-*C*. *burnetii* at 1:10000 in 0.1% Triton-X in PBS. Cells were washed with PBS, stained with DAPI and goat anti-rabbit IgG, Alexa Fluor 488 secondary antibody (Life Technologies, A11034). Foci were visualized by indirect immunofluorescence, manually counted and represented as FFU per ml of the primary infection sample by accounting for the dilution factor.

### qRT-PCR

*IDO1* expression in HeLa cells was measured by qRT-PCR using primers listed in Table 4. Fold increase in *IDO1* expression in IFNγ-treated, Mock or *IDO1* siRNA-transfected cells was calculated in comparison to untreated, mock transfected cells by assuming 100% efficiency for primers.

### Transmission electron microscopy

HeLa 229 cells were infected with wt *C*. *burnetii* at MOI 100 and left untreated or treated with IFNγ in the presence or absence of supplemented Trp at 6h pi. Cells were washed on d1 pi and Trp was supplemented in the appropriate samples. The following method was adapted based on published EM studies with *C*. *burnetii* [64] and recommendation from the Yale EM facility. On d4 pi, infected cells in petri dishes were fixed in 2.5% glutaraldehyde in 0.1M sodium cacodylate buffer pH7.4 containing 2% sucrose for 1h, then rinsed in buffer and replaced with 0.1% tannic acid in buffer for another hour. Buffer-rinsed cells were scraped in 1% gelatin and spun down in 2% agar. Chilled blocks were trimmed and post fixed in 1% osmium tetroxide and 1.5% potassium ferrocyanide in buffer for 1 hour. The samples were rinsed in sodium cacodylate and distilled water and en-bloc stained in aqueous 2% uranyl acetate for 1h. This was followed by further rinsing in distilled water and dehydrated through an ethanol series to 100%. The cells were infiltrated with Embed 812 (Electron Microscopy Sciences) resin, placed in silicone molds and baked at 60°C for 24h. Hardened blocks were sectioned using a Leica UltraCut UC7. 60nm sections were collected on formvar coated nickel grids and stained using 2% uranyl acetate and lead citrate. 60nm Grids were viewed FEI Tencai Biotwin TEM at 80Kv. Images were taken using Morada CCD and iTEM (Olympus) software. Images were resized and scale bars added using Adobe Illustrator.

### Inducible expression of IDO1 in HeLa cells

*IDO1* gene was cloned into an empty lentiviral vector pTRIPZ-EV under the tetracycline-inducible promoter at EcorI site by ligase independent cloning using the primers listed in Table 4. To derive the lentivirus, HEK293T cells were plated in 10cm dish and transfected with pTRIPZ-EV or pTRIPZ-*IDO1* with pVSV-G and psPAX2, as listed in Table 3, using Lipofectamine 2000 (Invitrogen). Lentiviral particles were obtained by collecting the supernatant at 48h and 72h post-transfection, pooled, filtered using 0.45μm low protein binding filter. Lentivirus containing pTRIPZ-EV or pTRIPZ-*IDO1* was used to transduce sub-confluent HeLa cells at half the volume of complete media, in the presence of polybrene (8μg/ml). Transduced cells were maintained in culture using puromycin (2.25μg/ml).

### Detection of IDO1 protein by immunoblot analysis

To test the efficiency of *IDO1* knockdown by siRNA, HeLa cells were plated and treated with *IDO1* siRNA and IFNγ 10ng/ml as described for the siRNA screen. 24h later, cells were washed and replenished with IFNγ-free media. Cells were collected two days post IFNγ treatment for immunoblotting. To test the expression of IDO1 in pTRIPZ-*IDO1* expressing HeLa cells, 10^5^ cells/w were plated in 24w plates and treated with increasing concentrations of anhydrotetracycline (referred to as Tet) or Doxycycline for 3 days or IFNγ 10ng/ml for 4 days. Cells treated with IFNγ were washed and replenished with IFNγ-free media 24h post-treatment. To determine if IDO1 is induced in THP1 cells, 4*10^5^ cells/w were plated in 12w plates and left untreated or differentiated with PMA overnight. Cells were washed and left untreated or treated with IFNγ 100ng/ml. IFNγ-treated cells were washed and replenished with IFNγ-free media after 24h. Cells were harvested 2 days post IFNγ-treatment. In all cases, cell lysates were prepared using Blue Loading Buffer (Cell signaling, #7722). Anti-IDO1 (BioLegend, W16073A), anti-α-tubulin (Sigma-Aldrich, T9026) prepared at 1:1000 in 5% BSA in 1X TBST and secondary antibodies goat anti-rat or anti-mouse IgG, HRP (Invitrogen, Cat. 62–9520 and 62–6520) were used to detect the proteins by western blotting.

### Data analysis and statistics

Data presented here are representative of at least 2 experiments. The siRNA screen (Fig 2) represents an average of 3 independent trials. Data were graphed and analyzed using Prism 7 software. Statistical significance was determined by t-test, one-way or two-way ANOVA depending on the number of groups and experimental conditions being compared.
